# Mesenchymal stem cell aggregation mediated by integrin α4/VCAM-1 after intrathecal transplantation in MCAO rats

**DOI:** 10.1186/s13287-022-03189-0

**Published:** 2022-10-22

**Authors:** Ye Ran, Yankai Dong, Yuejiao Li, Jundong Xie, Shubin Zeng, Chuanlei Liang, Wei Dai, Wenjing Tang, Yaojiong Wu, Shengyuan Yu

**Affiliations:** 1 Department of Neurology, The Chinese PLA General Hospital, Medical School of Chinese PLA, Beijing, 100853 China; 2 State Key Laboratory of Chemical Oncogenomics, Shenzhen Key Laboratory of Health Sciences and Technology, Institute of Biopharmaceutical and Health Engineering (iBHE), Shenzhen International Graduate School, Tsinghua University, Shenzhen, 518055 China; 3 School of Life Sciences, Tsinghua University, Beijing, 100084 China; 4 Tsinghua-Berkeley Shenzhen Institute (TBSI), Tsinghua University, Shenzhen, 518055 China

**Keywords:** Mesenchymal stem cell, Stroke, Cell aggregation, VCAM-1, Integrin α4

## Abstract

**Background:**

Mesenchymal stem cells (MSCs) have shown immense therapeutic potential for various brain diseases. Intrathecal administration of MSCs may enhance their recruitment to lesions in the central nervous system, but any impact on cerebrospinal fluid (CSF) flow remains unclear.

**Methods:**

Rats with or without middle cerebral artery occlusion (MCAO) received intrathecal injections of 2D cultured MSCs, 3D cultured MSCs or an equal volume of artificial cerebrospinal fluid (ACSF). Ventricle volume was assessed by MRI on Days 2 and 14 post-MCAO surgery. A beam walking test was used to assess fine motor coordination and balance. Aggregation of MSCs was evaluated in CSF and frozen brain tissue. Differential expression of cell adhesion molecules was evaluated by RNA-Seq, flow cytometry and immunofluorescence analyses. The influence of VCAM-1 blockade in mediating the aggregation of 2D MSCs was investigated in vitro by counting cells that passed through a strainer and in vivo by evaluating ventricular dilation.

**Results:**

MSC expanded in 2D culture formed aggregates in the CSF and caused ventricular enlargement in both MCAO and normal rats. Aggregates were associated with impaired motor function. 2D MSCs expressed higher levels of integrin α4 and VCAM-1 than 3D MSCs. Blockade of VCAM-1 in 2D MSCs reduced their aggregation in vitro and reduced lateral ventricular enlargement after intrathecal infusion. 3D MSCs exhibited lower cell aggregation and reduced cerebral ventricular dilation after intrathecal transplantation

**Conclusions:**

The aggregation of 2D MSCs, mediated by the interaction of integrin α4 and VCAM-1, is a potential risk for obstruction of CSF flow after intrathecal transplantation.

**Supplementary Information:**

The online version contains supplementary material available at 10.1186/s13287-022-03189-0.

## Introduction

Ischemic stroke is an important public health issue worldwide, with a high prevalence, disability rate, mortality rate and recidivism rate [1]. Although a variety of early recanalization treatments in clinical practice, such as intravenous thrombolysis and endovascular thrombectomy, can significantly benefit patients with ischemic stroke, the application of these methods has great limitations [2, 3]. No effective drugs are currently available to ameliorate sensorimotor function, resulting in most patients suffering from a lifelong disability. These facts illustrate the pressing need for novel therapeutic approaches to reduce neurological deficits.

Mesenchymal stem cells (MSCs) are multipotent stem cells, capable of differentiating into mesoderm- and nonmesoderm-derived tissues [4–6]. In addition to the potential for differentiation, MSCs secrete a variety of growth factors and cytokines known to enhance tissue repair/regeneration and regulate the immune response [7–9]. Increasing evidence of a profound therapeutic potential for MSCs for various diseases, including stroke, has emerged, and numerous clinical trials are underway [10–12]. However, MSCs cultured in 2D become trapped in the lungs after intravenous infusion, die quickly and are unable to reach extrapulmonary sites of damage [13–16]. Intra-arterial cell transplantation reduces cerebral blood flow and embolic events [17]. Therefore, local delivery of MSCs to target organs is a more efficient therapeutic approach [10, 18–20]. However, stereotactically guided neurosurgical cell delivery has the risk of inducing cerebral hemorrhage.

The intrathecal injection is a possible alternative route for stem cell delivery to the central nervous system (CNS) [21–23]. After injection into the subarachnoid space, MSCs are hypothesized to flow in the cerebrospinal fluid (CSF) and migrate into the CNS lesion. CSF is secreted by the choroid plexus, located within the ventricles, and flows through the median and lateral apertures to enter the subarachnoid cisterns. From there, it continues down through the subarachnoid space of the spinal cord and is absorbed into the venous system by arachnoid granulations. The rate of CSF secretion equals that of its removal, so obstruction of flow allows fluid accumulation and causes enlargement of the cavity in front of the blockage. Although several studies have demonstrated the feasibility of intrathecal transplantation of MSCs, the influence on CSF flow has not been carefully examined [22, 24, 25]. MSCs are known for their tendency to self-assemble and spontaneously form aggregates in suspensions [26, 27]. Combined with the slow rate of CSF flow, aggregation of MSCs has the potential to cause a severe obstruction of CSF flow.

In MSC culture, three-dimensional (3D) methods have attracted increasing attention, including scaffold-free hanging-drop systems [28], stirring cultures in spin flasks or rotating wall vessels [29], and membrane-fabricated cell aggregates [30]. It appears that cells in 3D culture form niches that differ from those in 2D culture by adhering to each other instead of sticking to the surface of the dish [31]. Our and others’ previous studies have shown that 3D culture of MSCs significantly reduced the cell volume by up to 70% [32, 33], increased the self-renewal and differentiation potency [29, 34, 35], improved the capacity of engraftment and homing [36], and increased the secretion of paracrine factors [36]. In addition, compared with 2D MSCs, 3D MSCs exhibited decreased expression of certain integrins that mediate cell adhesion to extracellular matrix molecules [37].

The current study examined the influence of MSCs on CSF flow and found that 2D MSCs formed cell aggregates after intrathecal injection, causing a ventricular enlargement in a rat model of ischemic stroke (middle cerebral artery occlusion, MCAO) and in normal rats. 3D MSCs reduced the expression of integrin α4 and VCAM-1 and caused less cell aggregation and ventricular dilation after intrathecal transplantation than 2D MSCs. The current findings suggest that the aggregation of 2D MSCs mediated by integrin α4 and VCAM-1 is a potential risk for obstruction of CSF flow after intrathecal transplantation.

## Materials and methods

### Animals

Male Sprague Dawley rats of 280–320 g body weight were obtained from the Laboratory Animal Center, Guangdong Province, China, and housed individually in a temperature- and humidity-controlled environment with free access to food and water and a 12 h light/12 h dark cycle with lights on at 07:00 a.m. The number of animals in each group was 6–8 under the premise of 4R (reduction, refinement, replacement, responsibility) principle in animal experiments, and statistical significance was ensured. A total of 94 rats were used. The number of rats in each experiment was indicated in figure legends. This study was approved by the Ethics Committee of Tsinghua Shenzhen International Graduate School, Tsinghua University, and guidelines on euthanasia and pain management were followed. The following measures were taken to reduce the peri-procedural pain of experimental animals: (1) The environment and surgical instruments were fully sterilized before surgery to reduce postoperative infection. (2) The use of isoflurane anesthesia with a high safety coefficient can avoid the pain of subcutaneous injection of anesthetics and adjust the concentration of anesthetics as needed to avoid pain in rats during severe pain stimulations, such as cutaneous suture, shorten recovery time from anesthesia after surgery, and reduce rat mortality. (3) After the operation, the rats were placed on the body heating plate and then transferred to the cage after they awoke to avoid hypothermia. (4) Before recovery from anesthesia, the rats were given carprofen (5 mg/kg) for pain relief, and then the drug was given every 24 h until 72 h after surgery. The veterinarian checked the pain, discomfort and wound of the experimental animals every day. (5) Avoid the presence of other rats while killing rats, so that animals do not suffer from fear and mental pain.

### Cell culture

MSCs were collected, with written consent from donors, and isolated from the human placenta, as previously described [27, 38]. The procedure was approved by the ethics committees of Peking University Shenzhen Hospital and Tsinghua University. Cells were expanded in 2D monolayer culture in Dulbecco’s modified Eagle’s medium (DMEM; Corning) supplemented with 10% fetal bovine serum (FBS; Biological Industries) at 37 ℃ with 5% CO_2_ in air. Cells were trypsinized and subcultured at 80% confluence. 3D spheroids were generated by culturing MSCs in a hanging drop as previously described [27, 39]. Briefly, MSCs at passages 5–7 were seeded in hanging drops (20,000 cells per drop) in 35 μl DMEM containing 10% FBS and incubated for 36 h. Spheroids were transferred to suspension culture and incubated in a fresh growth medium for 24 h. To obtain single cells from spheroids, spheroids were incubated with 0.25% trypsin/EDTA for 4–6 min with gentle pipetting every 2–3 min. MSCs were labeled with lentivirus green fluorescent protein (GFP; Cyagen), when indicated, according to the manufacturer’s instructions. MSCs at passages 5–6 before and after 3D culture were used for comparative analyses. The HaCaT cell line was purchased from iCell Bioscience Incorporated (Shanghai).

### Flow cytometry

The expression levels of cell surface proteins were determined by flow cytometry. Adherent cells were harvested by trypsin/EDTA (0.05%/0.02%), centrifuged, washed and resuspended in phosphate-buffered saline (PBS) containing 1% bovine serum albumin (BSA). In order to evaluate the expression of intercellular adhesion in MSCs, aliquots of 1.0 × 10^6^ cells were incubated with phycoerythrin (PE)-conjugated antibodies, CD49d (12–0499-73, eBioscience), CD106 (561679, BD Pharmingen) or PE antihuman CD324 (E-Cadherin; 324105, BioLegend) and corresponding isotype controls for 30 min before washing with PBS. Antibodies for MSC characterization were all purchased from BioLegend, which were fluorescein isothiocyanate (FITC)-conjugated monoclonal antibodies against CD45 (304005), CD34 (343503) and HLA-DR (307603) and allophycocyanin (APC)-conjugated antibodies against CD73 (344005), CD105 (endoglin; 323207) and CD90 (328113). All samples were analyzed by an EPICS XL flow cytometer (Beckman Coulter GmbH, Krefeld, Germany).

### MSC differentiation

MSCs in passage 5 were induced to differentiate into adipocytes, osteoblasts and chondrocytes as described in our previous publications [28, 38]. In adipogenic differentiation, MSCs were cultured in a medium containing 10^–6^ M dexamethasone, 10 µg/mL insulin, and 100 µg/mL 3-isobutyl-L-methylxantine (Sigma); after 3 weeks, the cells were stained with Oil Red-O to detect lipid. In osteogenic differentiation, the induction medium contained 10^–7^ M dexamethasone, 50 µg/ml ascorbic acid and 10 mMβ-glycerophosphate (Sigma), and the cultures were stained using Alizarin Red after 3 weeks. For chondrogenic differentiation, MSCs were cultured in DMEM (high glucose) containing 10^–7^ M dexamethasone, 50 µg/ml ascorbate-2-phosphate, 100 µg/ml pyruvate (Sigma), 10 ng/ml TGF-β1 (R&D Systems) and 50 mg/ml ITS + Premix (BD Biosciences, 6.25 µg/ml insulin, 6.25 µg/ml transferrin, 6.25 ng/ml selenious acid, 1.25 mg/ml bovine serum albumin and 5.35 mg/ml linoleic acid).

### RNA sequencing (RNA-Seq)

Total RNA was extracted from 2D MSCs at passage P5 and 3D spheroid MSCs prepared by culturing P5 2D MSCs as spheroids for 60 h. The mRNA was isolated using oligo(dT) magnetic beads and fragmented with a fragmentation buffer. First-strand cDNA was synthesized using a random hexamer primer, and then second-strand cDNA was synthesized. End reparation and 3’-end single nucleotide A (adenine) addition were performed. Sequencing adaptors were ligated to the fragments, and full-length cDNA was amplified by PCR. Library quality, quantity and average fragment sizes were assessed by an Agilent Bioanalyzer 2100. Rolling circle amplification (RCA) was performed to generate DNA nanoballs (DNBs) containing more than 300 copies of circular DNA. DNBs were loaded into a patterned nanoarray and single-end 50-base reads were generated by combinatorial probe-anchor synthesis (cPAS). Differentially expressed genes (DEGs) were identified by PossionDis analysis (false discovery rate < 0.001).

### Middle cerebral artery occlusion (MCAO) model

The Sprague–Dawley MCAO rat model was prepared as described previously [39]. In brief, rats were anesthetized with a mixture of 1–2% isoflurane in nitric oxide/oxygen via a face mask and placed on a heating pad to maintain body temperature. A ventral midline neck incision was made to expose the left common, internal and external carotid arteries. The common and external carotid arteries were ligated with 4–0 silk sutures. A blunt-end silicon-rubber-coated nylon monofilament was inserted into the internal carotid artery and forwarded into the middle cerebral artery origin. Reperfusion was achieved after 120 min of occlusion by slowly withdrawing the nylon monofilament. Sham animals underwent the same operation without occlusion.

### Intrathecal transplantation of MSCs

Male Sprague–Dawley rats (body weight 280–320 g) were randomly divided into three groups (*n* = 14) to receive intrathecal injections of 2D cultured MSCs, 3D cultured MSCs or an equal volume of artificial cerebrospinal fluid (ACSF, consisting of 126 mM NaCl, 26 mM NaHCO_3_, 3.0 mM KCl, 1.25 mM NaH_2_PO_4_, 2.0 mM CaCl_2_, 2 mM MgCl_2_ and 20 mM dextrose; 320 mOsm). Rats were anesthetized with a mixture of 1–2% isoflurane in nitric oxide/oxygen via a face mask and placed on a heating pad to maintain body temperature. A capillary tube connecting a 1-ml syringe through a polyethylene tube (0.5 mm internal diameter) was introduced into the cisterna magna through the dura mater. MSC single-cell suspensions were filtered through a cell strainer with 40 μm pores immediately before transplantation. Aliquots of 1 × 10^6^ 2D or 3D MSCs in 100 μl ACSF or an equal volume of ACSF, were slowly injected into the cisterna magna via the capillary tube over at least 5 min for MCAO rats (*n* = 8) on Day 2 and 7 postsurgery. Normal rats received 3 × 10^6^ 2D or 3D MSCs in 150 μl ACSF or an equal volume of ACSF into the cisterna magna (*n* = 6) to examine the influence of MSCs on CSF flow. MSCs were prelabeled with GFP and incubated with blocking antibody against VCAM-1 (R&D systems, AF809) or control isotype IgG at 30 μg/ml for 30 min at 4 °C prior to injection when indicated. MSCs were washed with PBS and filtered through a cell strainer with 40 μm pores immediately before injection.

### Magnetic resonance imaging (MRI)

A 7.0 T high-field small animal MRI system with a commercial rat coil (Biospin; Bruker, Billerica, MA. USA) was used. The repetition time for T2-weighted images was 2500 ms, and the echo time was 33 ms. Rats were anesthetized using 5% isoflurane in air in an anesthesia induction chamber and ~ 2.5% isoflurane in air maintained with a mask. Respiration and external body temperature were monitored during imaging using an MR-compatible small animal monitoring and gating system, and the temperature was maintained at 37 °C with a heating circulation bath. Cerebral infarction was assessed on Days 2 and 14 after MCAO surgery. Ventricle and infarct tissue volumes were outlined by hand on a T2-weighted MRI image with ImageJ software (version 2.0.0-rc-69/1.52p; National Institutes of Health, Bethesda, MD, USA). Infarct volume was calculated as infarct area × thickness (1.0 mm). The percentage of ventricular volume was calculated as follows: percentage cerebral ventricular volume = cerebral ventricular volume/cerebral hemisphere volume × 100%.

We used the relative value of the infarct or ventricular volume rather than the absolute value of the volume to reduce the bias caused by different brain sizes in different rats. Under the same basic conditions, the degree of cerebral edema and atrophy depends primarily on the volume of the infarct. On the second day after MCAO modeling, a brain MRI scan was performed on all rats, and then the rats successfully modeled were grouped by simple randomization using a random number table to ensure that the baseline volume of cerebral infarction in rats in different groups was constant, and the error caused by subsequent cerebral edema and atrophy was kept to a minimum. All assigned rats were included in the analysis. In addition, to facilitate comparison with other studies, raw data on infarct and ventricular volume are also provided in the Additional file 1.

### Behavioral test

Fine motor coordination and balance were assessed by the beam walking test. The apparatus consisted of an 80-cm-long beam of 1.2 cm width, resting on two stands 50 cm above a tabletop. Prior to the MCAO procedure, rats were trained to walk the beam three times a day for 2 days. The time taken to cross the beam and the number of paw slips (one or both feet coming off) during the process were recorded [40]. Data related to behavioral testing were acquired in a blinded manner.

### Cerebrospinal fluid cell count

Aliquots of 20 μl CSF were collected 3 days post-MSC transplantation, as previously described [41], and deposited on a glass slide. Samples were visualized under a fluorescence microscope and digitally scanned using an automatic scanning system (Slide Scan System SQD1000; TEKSQRAY, Shenzhen, China). The numbers of GFP-positive single and aggregated (3 or more cells) MSCs were counted using ImageJ software (ver. 1.52).

### Immunofluorescence

Rats were anesthetized with sodium pentobarbital (30 mg/kg, i.p.), and brain tissue was excised after perfusion with saline and sliced into 10-μm sections for freezing. Several consecutive sections of the entire brain were processed for immunofluorescent staining with DAPI before fluorescence microscopy. MSCs were immunostained following standard procedures to detect the expression of E-cadherin (13-1700, Invitrogen).

### Statistical analyses

Investigators conducting the experiment and analyzing the data were blind to the experimental group until the end of the study and all analyses. Statistical analysis was performed using R software (3.6.2). One-way ANOVA was used for numerical variables, followed by the Tukey’s HSD post hoc test for multiple comparisons. Wilcoxon rank sum tests were used for nonnormally distributed numerical data. A value of *p* < 0.05 was considered significant.

## Results

### Characterization of MSCs

**Fig. 1:**
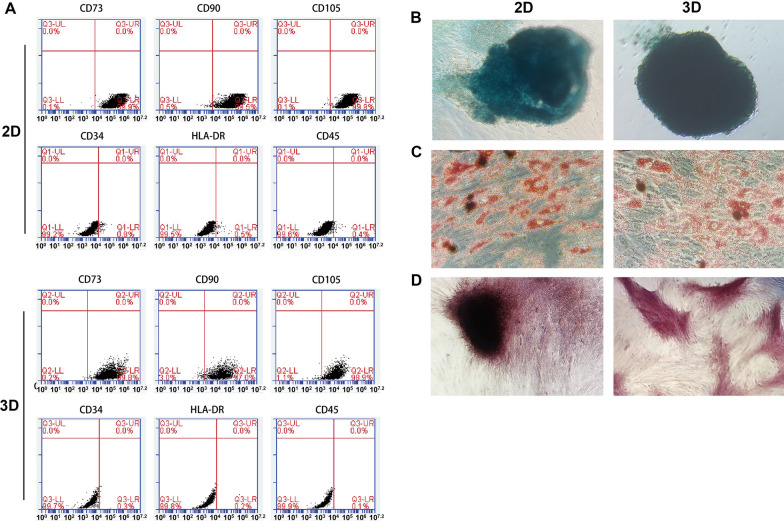
Characterization of MSCs. **A** Flow cytometry analysis of MSCs. MSCs at passage 5 before (2D) and after 3D culture were subjected to flow cytometry analysis for cell surface expression of different proteins as indicated. 3D cultured MSCs were dispersed into single cells by trypsinization before analysis. **B**–**D** Single-cell suspensions of 2D and 3D cultured MSCs were cultured in corresponding inductive media for chondrogenesis, adipogenesis and osteogenesis, for 14 days. In chondrogenic culture, cells tended to form aggregates, which were blue after Alcian Blue staining (**B**). In adipogenic culture, lipids in adipocytes were red after oil red staining (**C**). In osteogenic culture, mineral deposition was red after Alizarin Red S staining (**D**)

MSCs were characterized for their surface protein expression and differentiation potential using methods described in our previous publications [28, 38]. 2D cultured MSCs at passage 5 were examined before and after 3D culture. Flow cytometry analysis indicated that over 95% of 2D or 3D MSCs expressed CD73, CD90 and CD105, but less than 1% of the cells expressed CD45, CD34 and HLA-DR (MHC-II) (Fig. 1A). Upon culture in corresponding inductive media, both 2D cultured MSCs and 3D cultured MSCs were able to differentiate into adipocytes, osteoblasts and chondrocytes (Fig. 1B–D). The results indicate that our cells before and after 3D culture exhibit typical features of MSCs [42], consistent with our earlier findings [28].

### Ventricular enlargement occurs in MCAO rats receiving an intrathecal injection of MSCs

**Fig. 2:**
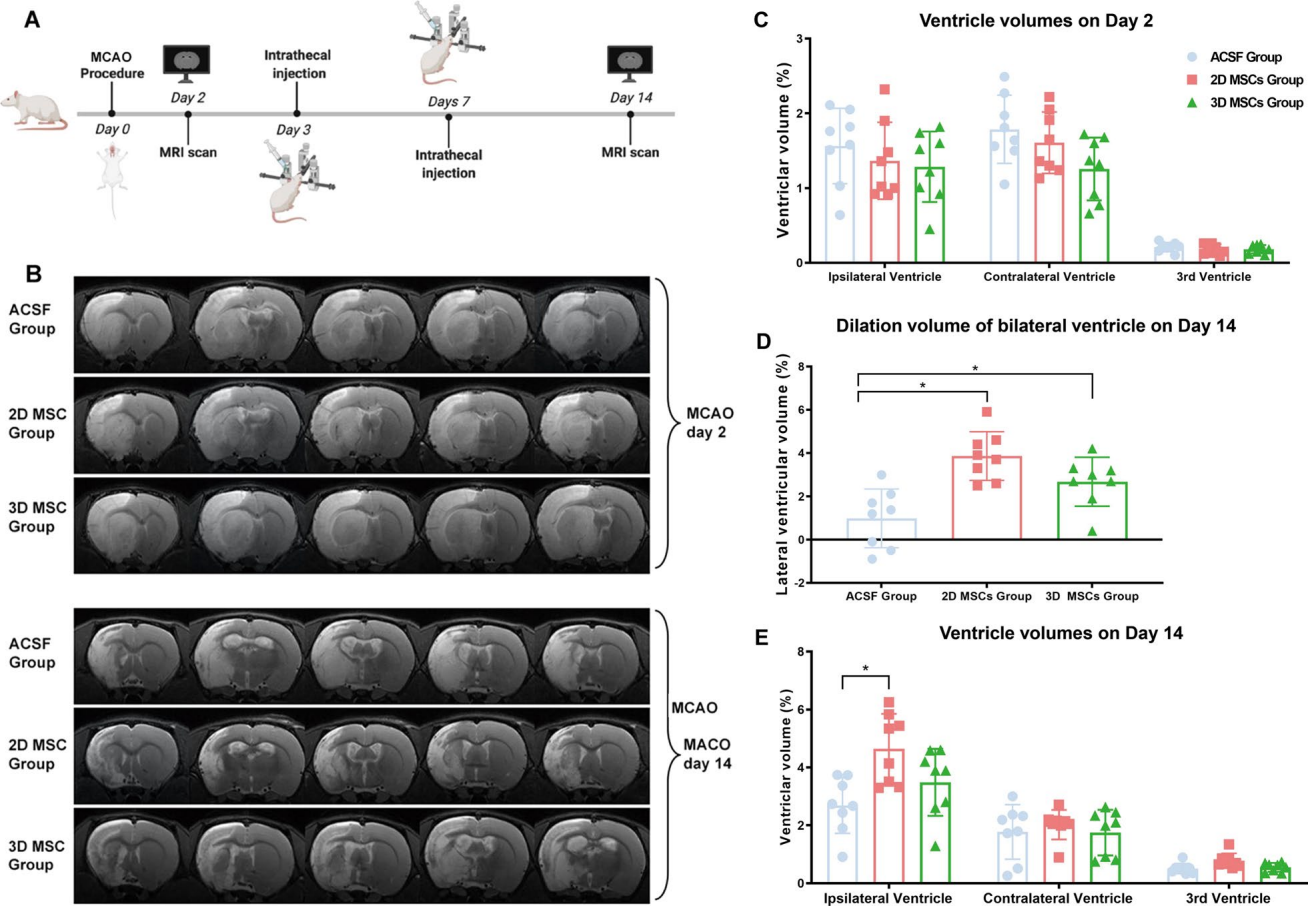
Ventricular enlargement in MCAO rats after intrathecal injection of MSCs. **A** Experimental scheme showing the times of MCAO surgery, MRI analysis and intrathecal injection of MSCs (1 × 10^6^ per dose) in rats. **B** MRI images showing the regions of cerebral infarcts and changes in lateral ventricular sizes before MSC transplantation (Day 2 post-MCAO) and after MSC transplantation (Day 14 post-MCAO; *n* = 8; representative images of one rat from each group are shown). **C**–**E** Measurement of ventricular sizes by MRI, indicated by ventricular volume as a percentage of bilateral hemisphere volume, showed no significant differences among the three groups on Day 2 before MSC transplantation (**C**). There was a significant increase in the size of the ipsilateral and the third ventricle in 2D MSC-treated rats but not in 3D MSC-treated rats, compared to ACSF-treated controls at 14 days (**D**; *P* < 0.05; *n* = 8). And the dilation volume of lateral ventricle in rats receiving MSCs was significantly higher than that in rats receiving ACSF (**E**; *P < 0.05; *n* = 8)

Rats with MCAO received an intrathecal injection of one million 2D cultured MSCs, 3D cultured MSCs or an equal volume of vehicle medium (ACSF) on Days 2 and 7 post-MCAO (Fig. 2A). Ventricular sizes were measured by MRI and are shown as the percentage of ventricular volume relative to the volume of the bilateral hemisphere. MRI analysis on Day 2 post-MCAO, prior to cell transplantation, showed no significant differences in ventricular size among the three groups (Fig. 2B, C). However, MRI results on Day 14 post-MCAO showed significant enlargement of the lateral ventricle in rats receiving both 2D (6.85 ± 1.04% hemispherical volume) and 3D (5.19 ± 1.45% hemispherical volume) MSCs compared with rats receiving ACSF (4.47 ± 1.83% hemispherical volume). No significant difference in volume between 2 and 3D MSC-treated rats was found although the enlargement appeared greater for the 2D MSC-treated rats (Fig. 2B, D). However, there was a significant increase in the size of the ipsilateral ventricle (4.64 ± 1.13% hemispherical volume) and third ventricle (0.78 ± 0.24% hemispherical volume) in 2D MSC-treated rats relative to ACSF-treated rats (2.69 ± 0.96% hemispherical volume and 0.51 ± 0.18% hemispherical volume, respectively) at 14 days post-MCAO which was not seen for the 3D MSC-treated rats (Fig. 2E; Additional file 1: Table S1).

### Intrathecal injection of MSCs caused a transient ventricular enlargement associated with impaired body balance

**Fig. 3:**
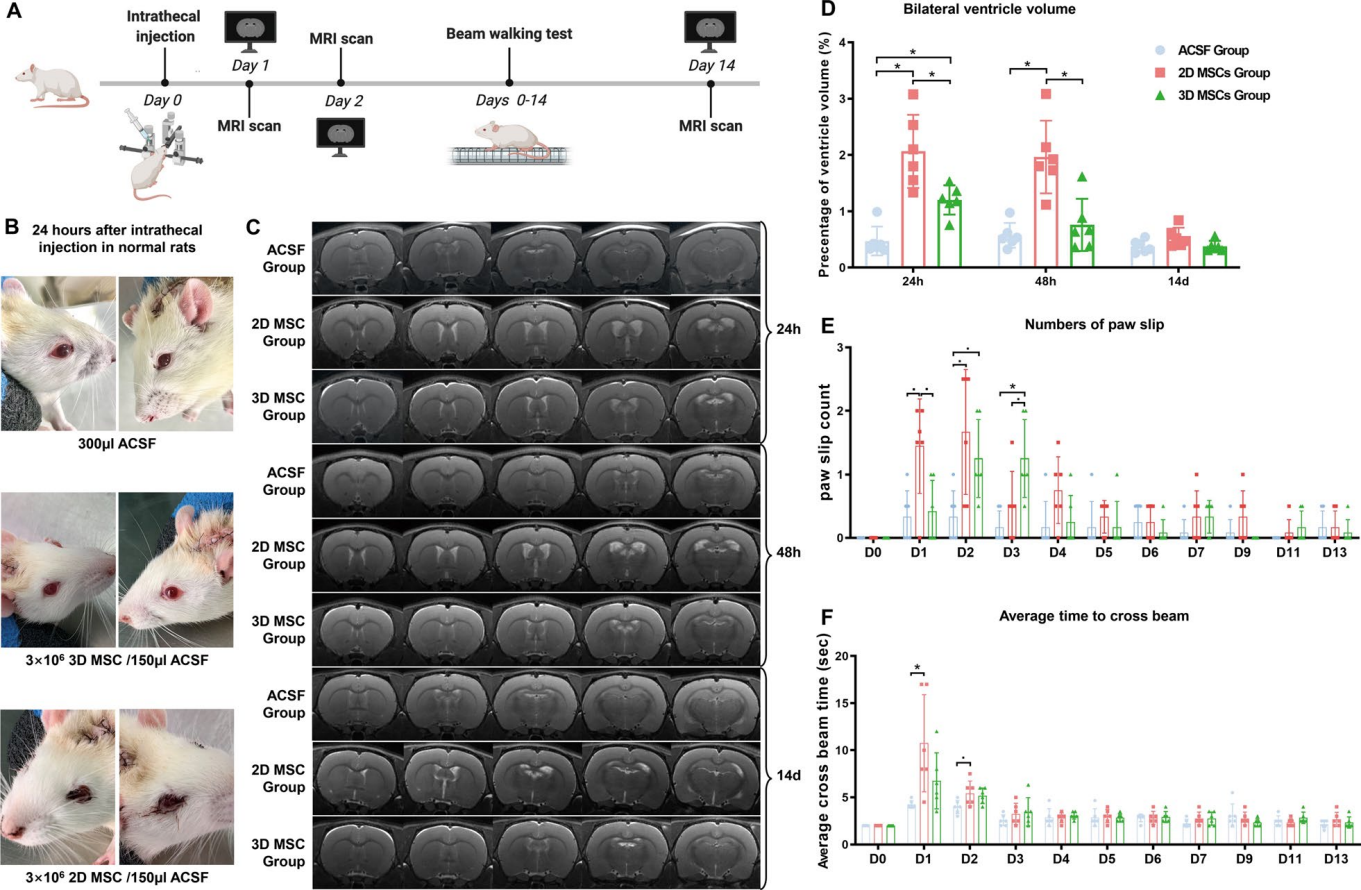
Influence of intrathecal MSC injection on ventricular volume and neurological function. **A** Experimental scheme showing the times of MRI scan, MSC injection (3 × 10^6^ per dose) and neurological functional analysis. **B** Twenty-four hours after injection, rats receiving 2D MSCs, but not 3D MSCs or ACSF, exhibited signs of blood congestion in the periorbital and perinasal regions that lasted several days (*n* = 6; representative images are shown). **C** MRI scans 24 h after MSC transplantation showed enlargements of the two lateral ventricles which was more evident in 2D MSC-treated rats (*n* = 6; representative images of one rat from each group are shown). **D** Measurement of ventricular volumes by MRI on Days 1, 2 and 14 post-transplantation of MSCs showed a decrease in the enlarged ventricle, particularly in rats receiving 3D MSCs (*P < 0.05; *n* = 6). **E**, **F** Beam walking analysis (number of paw slips) on different days (D1–13) following injection of MSCs with different treatments (**E**) and the average time taken to cross the beam (**F**) (*n* = 6; *P < 0.05, **·P** < 0.1)

Rats that had not undergone the MCAO procedure were given intrathecal infusions of 3 × 10^6^ MSCs to investigate whether the intrathecal injection, itself, had an impact on cerebral ventricle dilation (Fig. 3A). Within 24 h of injection, rats receiving 2D MSCs exhibited signs of blood congestion in the periorbital and perinasal regions which persisted for several days. Similar signs were not observed in rats injected with 3D MSCs or ACSF alone (Fig. 3B). MRI scans 24 h post-MSC transplantation showed enlargements of the two lateral ventricles relative to the controls (0.47 ± 0.23% hemispherical volume), which were more evident in 2D MSC-treated rats (2.07 ± 0.59% hemispherical volume) than in 3D MSC-treated rats (1.20 ± 0.24% hemispherical volume). Forty-eight hours after cell transplantation, MRI scans showed a modest decrease in the lateral ventricular volume in 2D MSC-treated rats (1.97 ± 0.59% hemispherical volume) and a sharp decrease in the lateral ventricular volume in 3D MSC-treated rats (0.76 ± 0.42% hemispherical volume), with the latter value being close to that for ACSF-treated controls (0.57 ± 0.20% hemispherical volume). Within 14 days, the ventricular volumes of both 2D (0.55 ± 0.15% hemispherical volume) and 3D (0.38 ± 0.09% hemispherical volume) MSC-treated rats had recovered to values similar to those seen for the ACSF group (0.38 ± 0.10% hemispherical volume; Fig. 3C, D; Additional file 1: Table S2).

Beam walking tests showed impairment in body balance in 2D MSC-treated rats which corresponded to changes in ventricular volumes. Twenty-four hours after transplantation, rats receiving 2D MSCs had more paw slip events (1.59 [1.50–1.92]) than controls (0.25 [0.00–0.50]) and significantly longer crossing times (9.00 [8.00–15.25] s vs. 4.00 [4.00–4.38] s), suggesting a deficit in motor function. The impairment lasted 2–3 days. There was no significant difference in crossing time between 3D MSC-treated rats and ACSF-treated rats but the former group did experience more paw slips in the first 3 days (Fig. 3E, F; Additional file 1: Tables S3, S4).

### 2D cultured MSCs form more abundant and larger aggregates in CSF

**Fig. 4:**
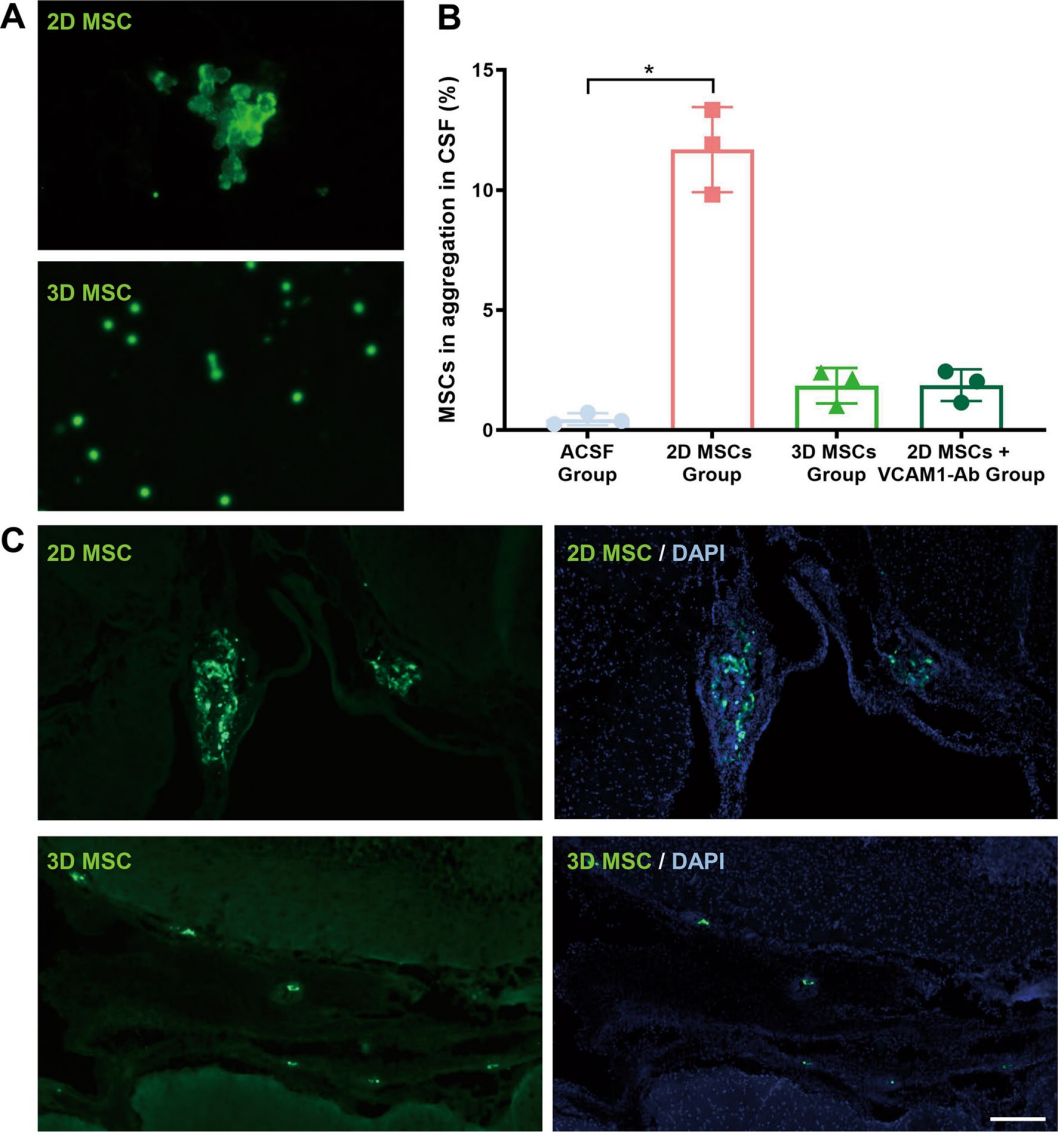
MSC tracing following intrathecal injection. **A**, **B** CSF of rats was collected 3 days after intrathecal injection of GFP-expressing MSCs and inspected under a fluorescence microscope. 2D MSCs formed more abundant and larger GFP^+^ cell aggregates in the CSF than 3D MSCs. Blockade of VCAM-1 in 2D MSCs reduced MSC aggregation. Samples from 3 rats in each group were analyzed, and representative images are shown (**A**). Average percentages of aggregated MSCs (≥ 3 cells per aggregate) were counted, including VCAM-1Ab group (**B**). **C** Brain sections. Three days post-MSC injection were examined under a fluorescence microscope. GFP^+^ cell aggregates were detected in the lateral ventricles of 2D MSC-treated rats, while single GFP^+^ cells were found in similar positions of 3D MSC-treated rats. Scale bar, 100 μm

Further investigations of MSC aggregate formation in CSF were conducted. CSF was collected 3 days after intrathecal injection of GFP-expressing MSCs and inspected under a fluorescence microscope. More abundant and larger GFP^+^ cell aggregates were seen in the CSF after the injection of 2D MSCs than after the injection of 3D MSCs (Fig. 4A, B; Additional file 1: Table S5). Brain tissue sections were examined 3 days after MSC injection under a fluorescence microscope. GFP^+^ cell aggregates were detected in the lateral ventricles of 2D MSC-treated rats, but only single GFP^+^ cells were present in the ventricles of 3D MSC-treated rats (Fig. 4C). These findings reinforce the view that 2D culture of MSCs makes the cells more prone to form aggregates in CSF.

### Differential expression of integrins and VCAM-1 in 2D and 3D cultured MSCs

**Fig. 5:**
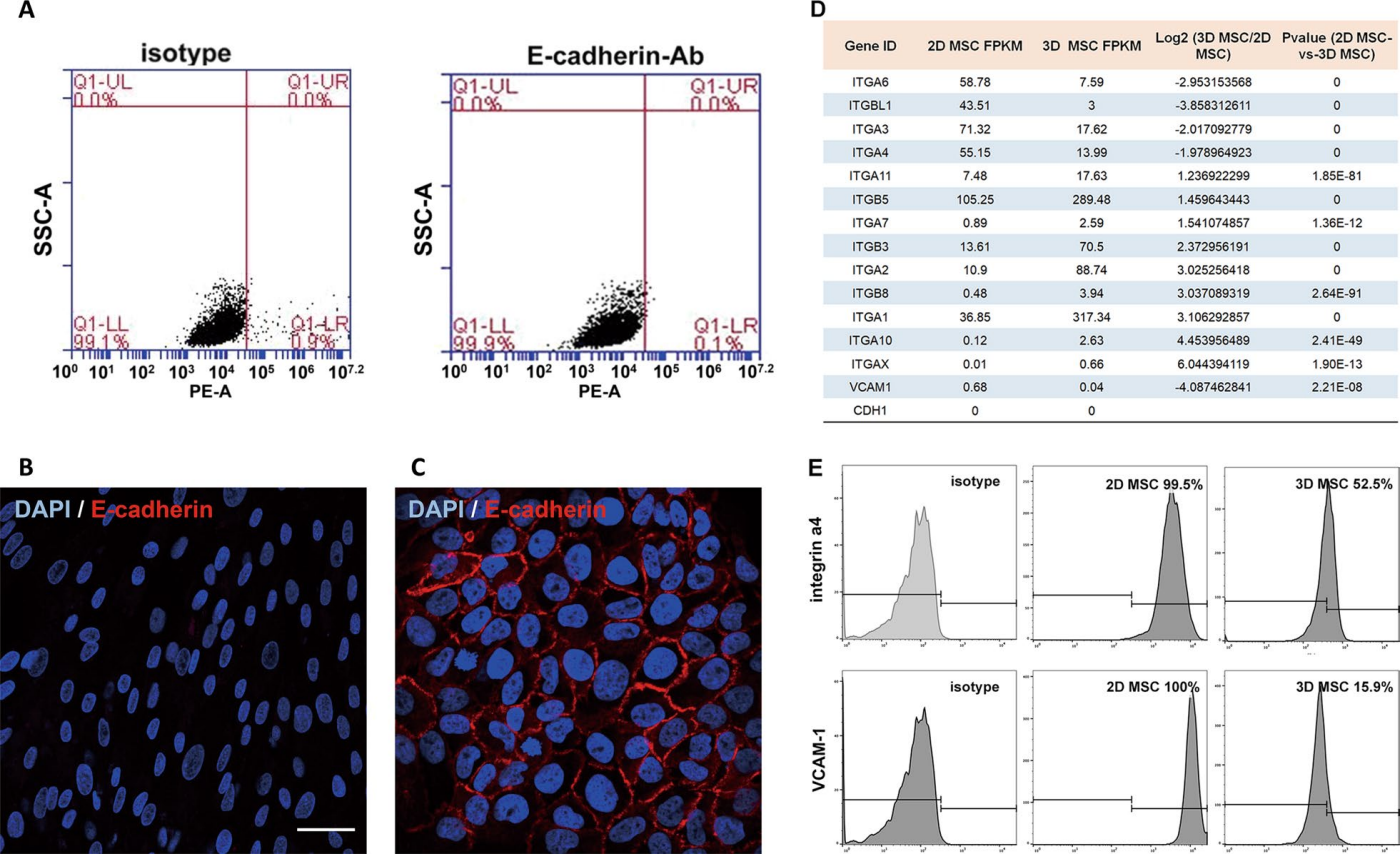
Expression of genes involved in intercellular adhesion in 2D versus 3D cultured MSCs. **A**, **B** Flow cytometry (**A**) and immunofluorescence (**B**) analyses of E-cadherin expression in 2D MSCs. **C** Skin epidermal cells (HaCaT) were used as a positive control for immunofluorescence analysis of E-cadherin. Scale bar, 50 μm (**B**, **C**). **D** Some differentially expressed genes involved in cell adhesion between 2D and 3D cultured MSCs were revealed by RNA-Seq analysis. **E** Flow cytometry analysis of 2D and 3D cultured MSCs for cell surface expression of integrin α4 and VCAM-1. Control cells were incubated with isotype IgG

The differential expression of cell adhesion genes, such as the gene encoding E-cadherin, a major protein for intercellular adhesion [43], in 2D- and 3D-cultured MSCs was investigated. E-cadherin expression could not be detected in 2D MSCs by flow cytometry or immunofluorescence analysis (Fig. 5A–C). Differential expression of several members of the integrin family of adhesion molecules was detected by RNA-Seq analysis. 3D MSCs showed lower levels of *Itgb1, Itga4, Itga6* and *Itga3* expression than 2D cells (Fig. 5D). In addition, the expression levels of integrin α4 and of the gene encoding its ligand, *vcam1*, were also lower. Integrin α4 mediates intercellular adhesion by forming a heterodimer with integrin β1 and binding VCAM-1. Such intercellular adhesion mechanisms are central to the formation of cell aggregates. 2D MSCs were found to express higher levels of cell surface integrin α4 and VCAM-1 than 3D MSCs by flow cytometry (Fig. 5E). Consistent results were derived from placental MSCs harvested from 3 different donors (data not shown).

### Blockade of VCAM-1 in 2D cultured MSCs decreased cell aggregation and ventricular dilation

**Fig. 6:**
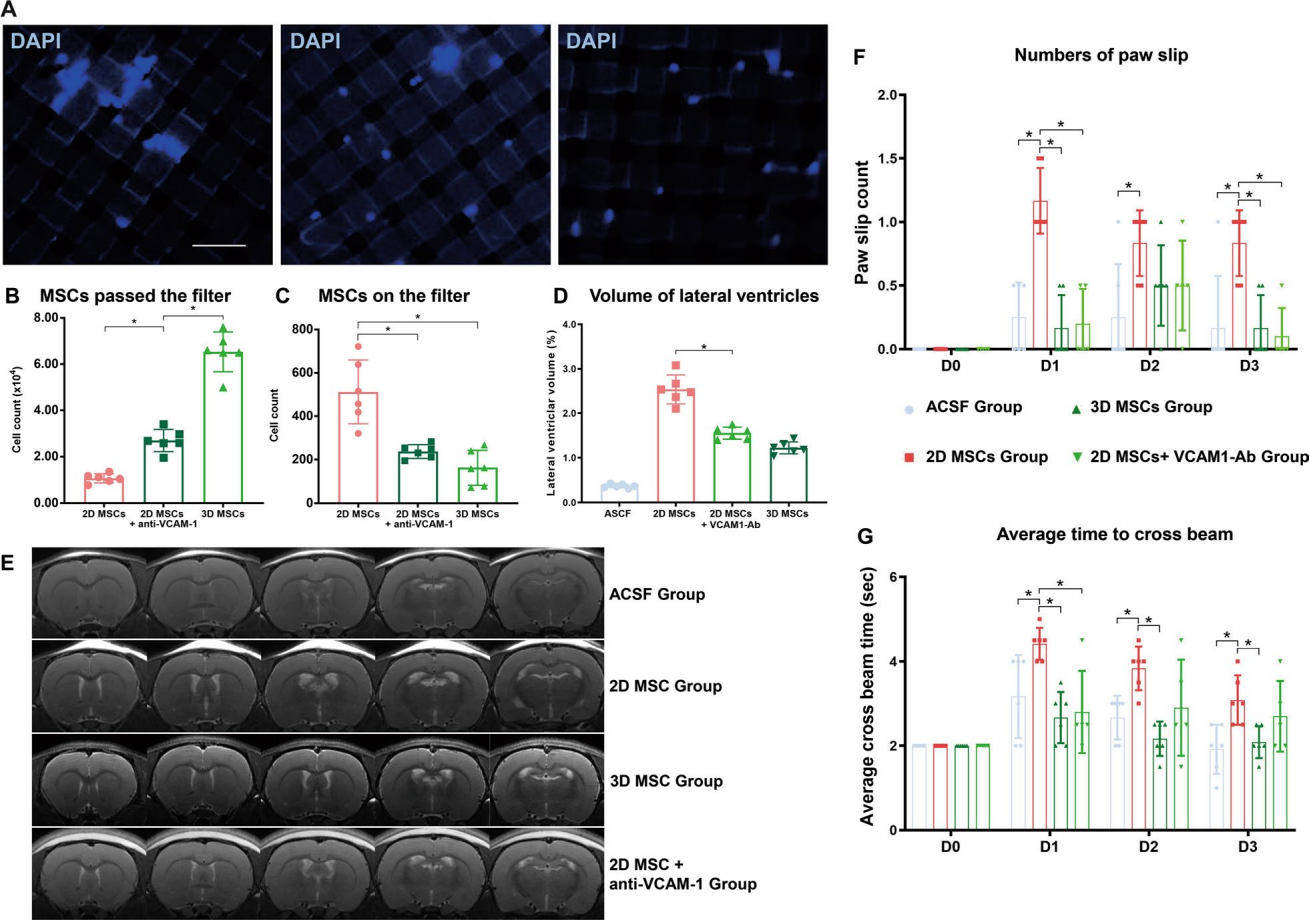
Influence of VCAM-1 blockade in 2D cultured MSCs on cell aggregation, ventricular dilation and neurological function. **A** Cell suspensions of 2D MSCs were incubated with an anti-VCAM-1 antibody or isotype IgG and 3D MSCs treated with isotype IgG served as a control. Cell suspensions were gently loaded on a cell strainer with 40 μm pores. Cells passing through the strainer were counted, and cells retained by the filter were stained with DAPI and visualized under a fluorescence microscope. Scale bar, 100 μm (**A**). The results showed considerably more 3D MSCs passing through the filter than 2D MSCs, and treatment of 2D MSCs with anti-VCAM-1 antibody increased the number of cells passing through the filter by over 60% (Fig. 5B). More 2D MSC aggregates were detected on the filter than 3D MSCs, and treatment of 2D MSCs with anti-VCAM-1 antibody reduced the number of aggregates on the filter (**A**, **C**; *n* = 6; *P < 0.05). **D**–**G** 2D MSCs incubated with anti-VCAM-1 antibody or isotype control were intrathecally injected. Rats injected with ASCF or 3D MSCs served as controls. After 24 h, MRI analysis was performed (**E**; *n* = 6; representative images of one rat from each group are shown). The bar plot shows a decrease in the enlarged ventricle in rats receiving 2D MSCs with anti-VCAM-1 antibody (**D**; *n* = 6; *P < 0.05). The number of paw slips (**F**) and the average time taken to cross the beam (**G**) were also determined by the beam walking test (*n* = 6; *P < 0.05)

The role of VCAM-1 in mediating MSC aggregation was examined in vitro. Cell suspensions of 2D MSCs were incubated with an antibody that blocked VCAM-1 function or with isotype IgG for 30 min at 37 °C. 3D MSCs were treated with isotype IgG as a control. Cell suspensions were gently loaded onto a cell strainer with 40 μm pores. Cells that passed through the strainer were counted, and cells retained by the filter were stained with DAPI and visualized under a fluorescence microscope (Fig. 6A). Considerably more 3D MSCs passed through the filter than 2D MSCs. Treatment of 2D MSCs with anti-VCAM-1 antibody increased the number of cells passing through the filter by over 60% (Fig. 6B). More 2D MSC aggregates remained on the filter than 3D MSCs aggregates, and treatment of 2D MSCs with anti-VCAM-1 antibody reduced the number of cells retained by the filter by over 50% (Fig. 6C).

The in vivo effect of VCAM-1 blockade on the aggregation of 2D MSCs in rat CSF after intrathecal injection was also examined. 2D MSCs were incubated with anti-VCAM-1 antibody prior to injection, resulting in a reduced number of aggregated MSCs in the CSF to a level comparable with that of 3D MSCs (Fig. 4B; Additional file 1: Table S6). A reduction in the enlargement of the lateral ventricular volume after VCAM-1 blockade of 2D MSCs 24 h after cell injection was also revealed by MRI (Fig. 6D, E). Moreover, the results of beam walking tests indicated that rats receiving 2D MSCs with VCAM-1 blockade exhibited fewer paw slips (Fig. 6F) and lower mean crossing times (Fig. 6G) than rats receiving 2D MSCs incubated with control isotype IgG (Additional file 1: Tables S7, S8, S9).

## Discussion

The intrathecal injection is generally considered an efficient route for CNS drug delivery and represents an attractive approach for MSC transplantation to treat CNS diseases. MSCs have the potential to flow through the CSF to reach lesions in the brain and spinal cord or to release bioactive factors into the CSF to facilitate healing. However, obstruction of CSF flow has long been a concern over this therapeutic approach given the known tendency for MSCs to form aggregates in suspension [26, 37].

The current study compared an MCAO rat model with normal rats to examine any impact of intrathecally injected MSCs on CSF flow. Intrathecal MSC injection induced transient ventricle dilation, particularly for 2D MSCs, and this dilation was associated with reduced balance. MSC aggregates were detected in the CSF and lateral ventricles. 2D MSCs tended to form more abundant and larger aggregates than 3D MSCs. MRI analysis showed that ventricle enlargement occurred within the first few days following MSC transplantation and recovered rapidly due to the death of aggregated MSCs. Notably, in rats with MCAO, the ventricular dilation after MSC transplantation lasted much longer, particularly in the injured hemisphere. The current findings thus confirm the risk of MSC aggregation and CSF obstruction after intrathecal transplantation. This research is well in line with STEPS recommendations [44–46].

Recent clinical and preclinical studies have established the safety of intrathecal MSC transplantation. Intrathecal infusion of 1.5 × 10^6^ BM-MSCs in dogs did not produce any differences in the number of nucleated cells or the levels of pro-MMP2 or MMP9 in the CSF before or after transplantation and MRI analysis one year post-transplantation did not reveal alterations in the brain [25]. Similarly, intrathecal infusion of 1.0 × 10^6^ BM-MSCs in horses did not produce changes in CSF cell count or pro-MMP2 level or cause signs of the neurological disorder [24]. Patients with chronic spinal cord injuries have also received intrathecal transplantation of human umbilical cord-derived MSCs. No serious side effects on the neurological function of a single injection of 10 × 10^6^ MSCs in 5 patients, where changes in cerebral ventricular size were not examined, were found at the 1- to 6-month follow-up [22]. Moreover, when 26 patients with amyotrophic lateral sclerosis received a single intrathecal dose of 15 × 10^6^ of BM-MSCs via lumbar puncture, although 30% experienced a mild to moderate headache, MRI examination of the brain and spinal cord at the 12-month follow-up did not detect new abnormal changes [47]. In the foregoing studies, changes in cerebral ventricle size were either not examined or examined months after MSC transplantation, at which time they were undetectable due to their transient nature. In addition, MSC dosages were much lower than the 1–2 million per kilogram body weight normally given via intravenous injection. The current results emphasize the need for dose escalation tests in large animals and patients to determine the safety of large or repeated doses of MSCs administered by intrathecal injection.

Mechanisms of MSC aggregation were also explored during this study. Several previous studies have shown that 2D MSCs tend to form aggregates in suspension [26, 37]. The mediator of intercellular adhesion, E-cadherin, was found to be expressed in cord tissue-derived MSCs and to contribute to MSC aggregation [48]. However, the current study found E-cadherin expression to be undetectable in placental MSCs. In contrast, 2D MSCs were found to express higher levels of integrin α4 and its binding partner, VCAM-1, than 3D MSCs. The interaction of integrin α4 and VCAM-1 is central to immune cell adhesion to inflamed endothelial cells [49, 50]. Indeed, overexpression of *ITGA4* in MSCs was found to enhance transendothelial migration in vitro, although a similar effect could not be reproduced in an in vivo rat model of stroke [51]. Blockade of VCAM-1 in 2D MSCs reduced cell aggregation in the present study. Moreover, intrathecal injection of 2D MSCs with VCAM-1 blockade reduced ventricular enlargement compared to control cells. These results indicate a critical role of integrin α4/VCAM-1 in MSC aggregation and CSF obstruction.

## Conclusion

To summarize, MSCs formed aggregates in CSF and caused ventricular dilation. The interaction of integrin α4 and VCAM-1 is likely to be central to MSC aggregation. 2D MSCs expressed higher levels of integrin α4 and VCAM-1, formed more abundant and larger aggregates in CSF and caused more severe ventricular enlargement than 3D MSCs. Our study provides insights into the design of MSC therapies for intrathecal transplantation.

## Supplementary Information

**Additional file 1.** Supplemental Tables.

## Data Availability

The datasets used and/or analyzed during the current study are available from the corresponding authors on reasonable request.

## Abbreviation

MSCs: Mesenchymal stem cells
CSF: Cerebrospinal fluid
MCAO: Middle cerebral artery occlusion
ACSF: Artificial cerebrospinal fluid
CNS: Central nervous system
DMEM: Dulbecco’s modified Eagle’s medium
FBS: Fetal bovine serum
GFP: Green fluorescent protein
PBS: Phosphate-buffered saline
BSA: Bovine serum albumin
PE: Phycoerythrin
RCA: Rolling circle amplification
DNBs: DNA nanoballs
cPAS: Combinatorial probe-anchor synthesis
DEGs: Differentially expressed genes
MRI: Magnetic resonance imaging

## References

[1] Feigin VL, Nguyen G, Cercy K, Johnson CO, Alam T, Parmar PG, Abajobir AA, Abate KH, Abd-Allah F, Abejie AN (2018). Global, regional, and country-specific lifetime risks of stroke, 1990 and 2016. New Engl J Med.

[2] Prabhakaran S, Ruff I, Bernstein RA (2015). Acute stroke intervention: a systematic review. JAMA.

[3] Zerna C, Thomalla G, Campbell BCV, Rha JH, Hill MD (2018). Current practice and future directions in the diagnosis and acute treatment of ischaemic stroke. Lancet.

[4] Prockop DJ (1997). Marrow stromal cells as stem cells for nonhematopoietic tissues. Science.

[5] Horwitz EM (2006). MSC: a coming of age in regenerative medicine. Cytotherapy.

[6] Wu Y, Chen L, Scott PG, Tredget EE (2007). Mesenchymal stem cells enhance wound healing through differentiation and angiogenesis. Stem Cells.

[7] Chen L, Tredget EE, Wu PY, Wu Y (2008). Paracrine factors of mesenchymal stem cells recruit macrophages and endothelial lineage cells and enhance wound healing. PLoS ONE.

[8] Wu Y, Zhao RC, Tredget EE (2010). Concise review: bone marrow-derived stem/progenitor cells in cutaneous repair and regeneration. Stem Cells.

[9] Shigemoto-Kuroda T, Oh JY, Kim DK, Jeong HJ, Park SY, Lee HJ, Park JW, Kim TW, An SY, Prockop DJ (2017). MSC-derived extracellular vesicles attenuate immune responses in two autoimmune murine models: type 1 diabetes and uveoretinitis. Stem Cell Rep.

[10] Mangi AA, Noiseux N, Kong D, He H, Rezvani M, Ingwall JS, Dzau VJ (2003). Mesenchymal stem cells modified with Akt prevent remodeling and restore performance of infarcted hearts. Nat Med.

[11] Squillaro T, Peluso G, Galderisi U (2016). Clinical trials with mesenchymal stem cells: an update. Cell Transplant.

[12] Guo Y, Peng Y, Zeng H, Chen G (2021). Progress in mesenchymal stem cell therapy for ischemic stroke. Stem Cells Int.

[13] Lee RH, Pulin AA, Seo MJ, Kota DJ, Ylostalo J, Larson BL, Semprun-Prieto L, Delafontaine P, Prockop DJ (2009). Intravenous hMSCs improve myocardial infarction in mice because cells embolized in lung are activated to secrete the anti-inflammatory protein TSG-6. Cell Stem Cell.

[14] Toma C, Wagner WR, Bowry S, Schwartz A, Villanueva F (2009). Fate of culture-expanded mesenchymal stem cells in the microvasculature: in vivo observations of cell kinetics. Circ Res.

[15] Ge J, Guo L, Wang S, Zhang Y, Cai T, Zhao RC, Wu Y (2014). The size of mesenchymal stem cells is a significant cause of vascular obstructions and stroke. Stem Cell Rev.

[16] Leibacher J, Henschler R (2016). Biodistribution, migration and homing of systemically applied mesenchymal stem/stromal cells. Stem Cell Res Ther.

[17] Cui LL, Kerkelä E, Bakreen A, Nitzsche F, Andrzejewska A, Nowakowski A, Janowski M, Walczak P, Boltze J, Lukomska B (2015). The cerebral embolism evoked by intra-arterial delivery of allogeneic bone marrow mesenchymal stem cells in rats is related to cell dose and infusion velocity. Stem Cell Res Ther.

[18] Karantalis V, DiFede DL, Gerstenblith G, Pham S, Symes J, Zambrano JP, Fishman J, Pattany P, McNiece I, Conte J (2014). Autologous mesenchymal stem cells produce concordant improvements in regional function, tissue perfusion, and fibrotic burden when administered to patients undergoing coronary artery bypass grafting. Circ Res.

[19] Sinder BP, Novak S, Wee NKY, Basile M, Maye P, Matthews BG, Kalajzic I (2020). Engraftment of skeletal progenitor cells by bone-directed transplantation improves osteogenesis imperfecta murine bone phenotype. Stem Cells.

[20] Liu Z, Mikrani R, Zubair HM, Taleb A, Naveed M, Baig M, Zhang Q, Li C, Habib M, Cui X (2020). Systemic and local delivery of mesenchymal stem cells for heart renovation: challenges and innovations. Eur J Pharmacol.

[21] Kim H, Na DL, Lee NK, Kim AR, Lee S, Jang H (2020). Intrathecal injection in a rat model: a potential route to deliver human wharton's jelly-derived mesenchymal stem cells into the brain. Int J Mol Sci.

[22] Albu S, Kumru H, Coll R, Vives J, Vallés M, Benito-Penalva J, Rodríguez L, Codinach M, Hernández J, Navarro X (2021). Clinical effects of intrathecal administration of expanded Wharton jelly mesenchymal stromal cells in patients with chronic complete spinal cord injury: a randomized controlled study. Cytotherapy.

[23] Boltze J, Arnold A, Walczak P, Jolkkonen J, Cui L, Wagner DC (2015). The dark side of the force—constraints and complications of cell therapies for stroke. Front Neurol.

[24] Maia L, da Cruz L-A, Taffarel MO, de Moraes CN, Machado GF, Melo GD, Amorim RM (2015). Feasibility and safety of intrathecal transplantation of autologous bone marrow mesenchymal stem cells in horses. BMC Vet Res.

[25] Benavides FP, Pinto GBA, Heckler MCT, Hurtado DMR, Teixeira LR, Monobe MMDS, Machado GF, de Melo GD, Rodríguez-Sánchez DN, Alvarenga FDCLE (2021). Intrathecal transplantation of autologous and allogeneic bone marrow-derived mesenchymal stem cells in dogs. Cell Transplant.

[26] Bartosh TJ, Ylostalo JH, Mohammadipoor A, Bazhanov N, Coble K, Claypool K, Lee RH, Choi H, Prockop DJ (2010). Aggregation of human mesenchymal stromal cells (MSCs) into 3D spheroids enhances their antiinflammatory properties. Proc Natl Acad Sci USA.

[27] Mo M, Zhou Y, Li S, Wu Y (2018). Three-dimensional culture reduces cell size by increasing vesicle excretion. Stem Cells.

[28] Guo L, Zhou Y, Wang S, Wu Y (2014). Epigenetic changes of mesenchymal stem cells in three-dimensional (3D) spheroids. J Cell Mol Med.

[29] Zhang S, Liu P, Chen L, Wang Y, Wang Z, Zhang B (2015). The effects of spheroid formation of adipose-derived stem cells in a microgravity bioreactor on stemness properties and therapeutic potential. Biomaterials.

[30] Huang GS, Dai LG, Yen BL, Hsu SH (2011). Spheroid formation of mesenchymal stem cells on chitosan and chitosan-hyaluronan membranes. Biomaterials.

[31] Marx V (2013). Cell culture: a better brew. Nature.

[32] Ge J, Guo L, Wang S, Zhang Y, Cai T, Zhao RC, Wu Y (2014). The size of mesenchymal stem cells is a significant cause of vascular obstructions and stroke. Stem Cell Rev Rep.

[33] Zhou Y, Chen H, Li H, Wu Y (2017). 3D culture increases pluripotent gene expression in mesenchymal stem cells through relaxation of cytoskeleton tension. J Cell Mol Med.

[34] Cheng NC, Wang S, Young TH (2012). The influence of spheroid formation of human adipose-derived stem cells on chitosan films on stemness and differentiation capabilities. Biomaterials.

[35] Wang W, Itaka K, Ohba S, Nishiyama N, Chung UI, Yamasaki Y, Kataoka K (2009). 3D spheroid culture system on micropatterned substrates for improved differentiation efficiency of multipotent mesenchymal stem cells. Biomaterials.

[36] Bartosh TJ, Ylöstalo JH, Mohammadipoor A, Bazhanov N, Coble K, Claypool K, Lee RH, Choi H, Prockop DJ (2010). Aggregation of human mesenchymal stromal cells (MSCs) into 3D spheroids enhances their antiinflammatory properties. Proc Natl Acad Sci USA.

[37] Wang S, Guo L, Ge J, Yu L, Cai T, Tian R, Jiang Y, Zhao R, Wu Y (2015). Excess integrins cause lung entrapment of mesenchymal stem cells. Stem Cells.

[38] Li Z, Liu C, Xie Z, Song P, Zhao RC, Guo L, Liu Z, Wu Y (2011). Epigenetic dysregulation in mesenchymal stem cell aging and spontaneous differentiation. PLoS ONE.

[39] Guo L, Ge J, Zhou Y, Wang S, Zhao RC, Wu Y (2014). Three-dimensional spheroid-cultured mesenchymal stem cells devoid of embolism attenuate brain stroke injury after intra-arterial injection. Stem Cells Dev.

[40] Luong TN, Carlisle HJ, Southwell A, Patterson PH (2011). Assessment of motor balance and coordination in mice using the balance beam. J Vis Exp.

[41] Maia LF, Kaeser SA, Reichwald J, Hruscha M, Martus P, Staufenbiel M, Jucker M (2013). Changes in amyloid-beta and Tau in the cerebrospinal fluid of transgenic mice overexpressing amyloid precursor protein. Sci Transl Med.

[42] Dominici M, Le Blanc K, Mueller I, Slaper-Cortenbach I, Marini F, Krause D, Deans R, Keating A, Prockop D, Horwitz E (2006). Minimal criteria for defining multipotent mesenchymal stromal cells: the International Society for Cellular Therapy position statement. Cytotherapy.

[43] Gumbiner BM (2005). Regulation of cadherin-mediated adhesion in morphogenesis. Nat Rev Mol Cell Biol.

[44] Boltze J, Modo MM, Mays RW, Taguchi A, Jolkkonen J, Savitz SI (2019). Stem cells as an emerging paradigm in stroke 4: advancing and accelerating preclinical research. Stroke.

[45] Savitz SI, Chopp M, Deans R, Carmichael T, Phinney D, Wechsler L (2011). Stem cell therapy as an emerging paradigm for stroke (STEPS) II. Stroke.

[46] STEPS Participants. Stem cell therapies as an emerging paradigm in stroke (STEPS): bridging basic and clinical science for cellular and neurogenic factor therapy in treating stroke. Stroke. 2009;40(2):510–515. 10.1161/STROKEAHA.108.526863 19095993

[47] Syková E, Rychmach P, Drahorádová I, Konrádová Š, Růžičková K, Voříšek I, Forostyak S, Homola A, Bojar M (2017). Transplantation of mesenchymal stromal cells in patients with amyotrophic lateral sclerosis: results of phase I/IIa clinical trial. Cell Transplant.

[48] Lee EJ, Park SJ, Kang SK, Kim GH, Kang HJ, Lee SW, Jeon HB, Kim HS (2012). Spherical bullet formation via E-cadherin promotes therapeutic potency of mesenchymal stem cells derived from human umbilical cord blood for myocardial infarction. Mol Ther.

[49] Worthylake RA, Burridge K (2001). Leukocyte transendothelial migration: orchestrating the underlying molecular machinery. Curr Opin Cell Biol.

[50] Elices MJ, Osborn L, Takada Y, Crouse C, Luhowskyj S, Hemler ME, Lobb RR (1990). VCAM-1 on activated endothelium interacts with the leukocyte integrin VLA-4 at a site distinct from the VLA-4/fibronectin binding site. Cell.

[51] Cui L-l, Nitzsche F, Pryazhnikov E, Tibeykina M, Tolppanen L, Rytkönen J, Huhtala T, Mu J-W, Khiroug L, Boltze J (2017). Integrin a4 overexpression on rat mesenchymal stem cells enhances transmigration and reduces cerebral embolism after intracarotid injection. Stroke.

