# Supplementary material for: Mesenchymal stem cell aggregation mediated by integrin α4/VCAM-1 after intrathecal transplantation in MCAO rats
Source: Stem Cell Res Ther. 2022 Oct 22;13:507. doi: 10.1186/s13287-022-03189-0 (PMC9587602; doi:10.1186/s13287-022-03189-0)
Supplement: Supplementary file 1 — Additional file 1. Supplemental Tables. [file 13287_2022_3189_MOESM1_ESM.docx]

**Supplemental Table 1. Volume of each ventricle in MCAO rats after intrathecal injection of MSCs (n=8)**

|  |  | ACSF group | 2D MSCs group | 3D MSCs Group |
| --- | --- | --- | --- | --- |
| 2 days  after MCAO | Ipsilateral Ventricle (mean ± SD) | 1.56 ± 0.55%  1.51 ± 0.51 mm^3^ | 1.37 ± 0.52%  1.28 ± 0.48 mm^3^ | 1.29 ± 0.47%  1.32 ± 0.53 mm^3^ |
|  | Contralateral Ventricle  (mean ± SD) | 1.97 ± 0.65%  1.73 ± 0.46 mm^3^ | 1.61 ± 0.41%  1.50 ± 0.40 mm^3^ | 1.25 ± 0.42%  1.29 ± 0.47 mm^3^ |
|  | 3rd Ventricle  (mean ± SD) | 0.22 ± 0.06%  0.21 ± 0.05 mm^3^ | 0.18 ± 0.07%  0.17 ± 0.06 mm^3^ | 0.18 ± 0.06%  0.19 ± 0.06 mm^3^ |
| 14 days after MCAO | Ipsilateral Ventricle (mean ± SD) | 2.69 ± 1.04%  2.61 ± 0.97 mm^3^ | 4.64 ± 1.21%  4.31 ± 1.06 mm^3^ | 3.48 ± 1.16%  3.59 ± 1.36 mm^3^ |
|  | Contralateral Ventricle  (mean ± SD) | 1.77 ± 1.02%  1.73 ± 0.93 mm^3^ | 2.02 ± 0.51%  1.90 ± 0.48 mm^3^ | 1.75 ± 0.79%  1.80 ± 0.87 mm^3^ |
|  | 3rd Ventricle  (mean ± SD) | 0.51 ± 0.19%  0.49 ± 0.17 mm^3^ | 0.78 ± 0.26%  0.72 ± 0.23 mm^3^ | 0.55 ± 0.16%  0.56 ± 0.19 mm^3^ |
| MSCs: mesenchymal stem cells; ACSF: artificial cerebrospinal fluid; MCAO: middle cerebral artery occlusion; SD: standard deviation.  The percentage of ventricular volume was calculated as follows: percentage cerebral ventricular volume = cerebral ventricular volume/cerebral hemisphere volume × 100%. | | | | |

**Supplemental Table 2. Volume of lateral ventricles in normal rats after intrathecal injection of MSCs (n=6)**

|  | ACSF group | 2D MSCs group | 3D MSCs Group |
| --- | --- | --- | --- |
| 24 hours after MCAO  (mean ± SD) | 0.47 ± 0.26%  0.87 ± 0.45 mm^3^ | 2.07 ± 0.65%  3.97 ± 1.24 mm^3^ | 1.20 ± 0.26%  2.31 ± 0.48 mm^3^ |
| 48 hours after MCAO  (mean ± SD) | 0.57 ± 0.22%  1.06 ± 0.39 mm^3^ | 1.97 ± 0.65%  3.79 ± 1.29 mm^3^ | 0.76 ± 0.42%  1.46 ± 0.88 mm^3^ |
| 14 days after MCAO  (mean ± SD) | 0.38 ± 0.11%  0.70 ± 0.20 mm^3^ | 0.54 ± 0.17%  1.04 ± 0.33 mm^3^ | 0.38 ± 0.09%  0.73 ± 0.17 mm^3^ |
| MSCs: mesenchymal stem cells; ACSF: artificial cerebrospinal fluid; MCAO: middle cerebral artery occlusion; SD: standard deviation. The percentage of ventricular volume was calculated as follows: percentage cerebral ventricular volume = cerebral ventricular volume/cerebral hemisphere volume × 100%. | | | |

**Supplemental Table 3. Paw slip count of rats after intrathecal injection of MSCs (n=6)**

|  | ACSF group | 2D MSCs group | 3D MSCs Group |
| --- | --- | --- | --- |
| Day 0 | 0.00 [0.00, 0.00] | 0.00 [0.00, 0.00] | 0.00 [0.00, 0.00] |
| Day 1 | 0.25 [0.00, 0.50] | 1.59 [1.50, 1.92] | 0.25 [0.00, 0.88] |
| Day 2 | 0.25 [0.00, 0.50] | 2.00 [0.75, 2.50] | 1.00 [1.00, 1.75] |
| Day 3 | 0.00 [0.00, 0.38] | 0.50 [0.13, 0.50] | 0.67 [1.00, 1.75] |
| Day 4 | 0.00 [0.00, 0.00] | 0.75 [0.50, 1.00] | 0.00 [0.00, 0.38] |
| Day 5 | 0.00 [0.00, 0.00] | 0.50 [0.13, 0.50] | 0.00 [0.00, 0.00] |
| Day 6 | 0.25 [0.00, 0.50] | 0.25 [0.00, 0.50] | 0.00 [0.00, 0.00] |
| Day 7 | 0.00 [0.00, 0.00] | 0.25 [0.00, 0.50] | 0.50 [0.50, 0.50] |
| Day 9 | 0.00 [0.00, 0.00] | 0.25 [0.00, 0.50] | 0.00 [0.00, 0.00] |
| Day 11 | 0.00 [0.00, 0.00] | 0.00 [0.00, 0.00] | 0.00 [0.00, 0.38] |
| Day 13 | 0.00 [0.00, 0.38] | 0.00 [0.00, 0.38] | 0.00 [0.00, 0.00] |
| Paw slip count (median [quartile 1, quartile 3])  MSCs: mesenchymal stem cells; ACSF: artificial cerebrospinal fluid. | | | |

**Supplemental Table 4. Cross beam time of rats after intrathecal injection of MSCs (n=6)**

|  | ACSF group | 2D MSCs group | 3D MSCs Group |
| --- | --- | --- | --- |
| Day 0 | 2.00 [2.00, 2.00] | 2.00 [2.00, 2.00] | 2.00 [2.00, 2.00] |
| Day 1 | 4.00 [4.00, 4.38] | 9.00 [8.00, 15.25] | 6.00 [5.13, 7.63] |
| Day 2 | 4.00 [3.63, 4.38] | 5.25 [4.50, 6.00] | 5.25 [4.63, 5.88] |
| Day 3 | 2.50 [2.13, 2.88] | 3.00 [2.50, 3.88] | 3.25 [2.63, 3.50] |
| Day 4 | 2.50 [2.50, 2.88] | 3.00 [3.63, 3.00] | 3.00 [3.00, 3.38] |
| Day 5 | 2.50 [2.50, 2.88] | 3.00 [2.63, 3.38] | 2.75 [2.50, 3.00] |
| Day 6 | 3.00 [3.00, 3.00] | 2.75 [2.50, 3.00] | 2.75 [2.50, 3.00] |
| Day 7 | 2.00 [2.00, 2.38] | 2.50 [2.50, 2.88] | 2.75 [2.13, 3.38] |
| Day 9 | 2.75 [2.50, 3.00] | 2.50 [2.50, 2.88] | 2.25 [2.00, 2.50] |
| Day 11 | 2.50 [2.13, 2.50] | 2.25 [2.00, 2.50] | 2.50 [2.50, 2.88] |
| Day 13 | 2.25 [2.00, 2.50] | 2.50 [2.13, 2.88] | 2.00 [2.00, 2.38] |
| Cross beam time (sec) (median [quartile 1, quartile 3])  MSCs: mesenchymal stem cells; ACSF: artificial cerebrospinal fluid. | | | |

**Supplemental Table 5. MSCs in aggregation in CSF (n=6)**

|  | ACSF  group | 2D MSCs group | 3D MSCs Group | 2D MSCs + VCAM1 ab Group |
| --- | --- | --- | --- | --- |
| Aggregation  rate (mean ± SD %) | 0.45 ± 0.24% | 11.68 ± 1.78% | 1.85 ± 0.74% | 1.87 ± 0.66% |
| MSCs: mesenchymal stem cells; ACSF: artificial cerebrospinal fluid; VCAM1-ab: anti-VCAM-1 antibody; SD: standard deviation. | | | | |

**Supplemental Table 6. MSCs in aggregation in vitro (n=6)**

|  | 2D MSCs group | 3D MSCs Group | 2D MSCs + VCAM1-ab Group |
| --- | --- | --- | --- |
| MSCs passed the filter (×10^4^) (mean ± SD) | 1.07 ± 0.19 | 6.53 ± 0.86 | 2.70 ± 0.48 |
| MSCs on the filter  (mean ± SD) | 512.33 ± 147.24 | 163.33 ± 80.17 | 237.50 ± 31.61 |
| MSCs: mesenchymal stem cells; ACSF: artificial cerebrospinal fluid; VCAM1-ab: anti-VCAM-1 antibody; SD: standard deviation. | | | |

**Supplemental Table 7. Volume of lateral ventricles in normal rats after intrathecal injection of ACSF, 2D MSCs, 3D MSCs, and 2D MSCs incubated with anti-VCAM-1 antibody (n=6)**

|  | ACSF  Group | 2D MSCs  Group | 3D MSCs  Group | 2D MSCs + VCAM1-ab Group |
| --- | --- | --- | --- | --- |
| 24 hours after intrathecal injection  (mean ± SD) | 0.37 ± 0.04%  0.69 ± 0.09 mm^3^ | 2.53 ± 0.33%  4.81 ± 0.66 mm^3^ | 1.23 ± 0.06%  2.34 ± 0.21 mm^3^ | 1.55 ± 0.07%  2.95 ± 0.24 mm^3^ |
| MSCs: mesenchymal stem cells; ACSF: artificial cerebrospinal fluid; VCAM1-ab: anti-VCAM-1 antibody; SD: standard deviation.  The percentage of ventricular volume was calculated as follows: percentage cerebral ventricular volume = cerebral ventricular volume/cerebral hemisphere volume × 100%. | | | | |

**Supplemental Table 8. Paw slip of beam walking test in normal rats after intrathecal injection of ACSF, 2D MSCs, 3D MSCs, and 2D MSCs incubated with anti-VCAM-1 antibody (n=6)**

|  | ACSF Group | 2D MSCs Group | 3D MSCs Group | 2D MSCs + VCAM1-ab Group |
| --- | --- | --- | --- | --- |
| Day 0 | 0.00 [0.00, 0.00] | 0.00 [0.00, 0.00] | 0.00 [0.00, 0.00] | 0.00 [0.00, 0.00] |
| Day 1 | 0.25 [0.00, 0.50] | 1.00 [1.00, 1.38] | 0.00 [0.00, 0.38] | 0.00 [0.00, 0.38] |
| Day 2 | 0.00 [0.00, 0.38] | 1.00 [0.63, 1.00] | 0.50 [0.50, 0.50] | 0.50 [0.50, 0.50] |
| Day 3 | 0.00 [0.00, 0.00] | 0.75 [0.50, 1.00] | 0.00 [0.00, 0.38] | 0.00 [0.00, 0.00] |
| Paw slip count (median [quartile 1, quartile 3])  MSCs: mesenchymal stem cells; ACSF: artificial cerebrospinal fluid;  VCAM1-ab: anti-VCAM-1 antibody. | | | | |

**Supplemental Table 9. Cross beam time in normal rats after intrathecal injection of ACSF, 2D MSCs, 3D MSCs, and 2D MSCs incubated with anti-VCAM-1 antibody (n=6)**

|  | ACSF Group | 2D MSCs Group | 3D MSCs Group | 2D MSCs + VCAM1-ab Group |
| --- | --- | --- | --- | --- |
| Day 0 | 2.00 [2.00, 2.00] | 2.00 [2.00, 2.00] | 2.00 [2.00, 2.00] | 2.00 [2.00, 2.00] |
| Day 1 | 3.50 [2.25, 4.00] | 4.50 [4.13, 4.50] | 2.75 [2.13, 3.00] | 2.50 [2.50, 2.50] |
| Day 2 | 3.00 [2.25, 3.00] | 4.00 [3.63, 4.00] | 2.25 [2.00, 2.50] | 2.75 [2.50, 3.38] |
| Day 3 | 2.00 [1.63, 2.38] | 3.00 [2.63, 3.38] | 2.00 [2.00, 2.38] | 2.50 [2.13, 2.88] |
| Cross beam time (sec) (median [quartile 1, quartile 3])  MSCs: mesenchymal stem cells; ACSF: artificial cerebrospinal fluid; VCAM1-ab: anti-VCAM-1 antibody. | | | | |
